# Automated Trimethyl Sulfonium Hydroxide Derivatization Method for High-Throughput Fatty Acid Profiling by Gas Chromatography–Mass Spectrometry

**DOI:** 10.3390/molecules26206246

**Published:** 2021-10-15

**Authors:** Paul Gries, Atul Singh Rathore, Xiyuan Lu, Jennifer Chiou, Yen Bao Huynh, Alessia Lodi, Stefano Tiziani

**Affiliations:** 1 Department of Nutritional Sciences, College of Natural Sciences, The University of Texas at Austin, Austin, TX 78712, USA; gries.paul@utexas.edu (P.G.); atulsr2001@utexas.edu (A.S.R.); xlu@utexas.edu (X.L.); jennifer.chiou@austin.utexas.edu (J.C.); yen.hby@utexas.edu (Y.B.H.); alessialodi@utexas.edu (A.L.); 2 Department of Pediatrics, Dell Medical School, The University of Texas at Austin, Austin, TX 78723, USA; 3 Department of Oncology, Dell Medical School, LiveSTRONG Cancer Institutes, The University of Texas at Austin, Austin, TX 78723, USA

**Keywords:** GC–MS, fatty acid profiling, online automated derivatization, trimethyl sulfonium hydroxide

## Abstract

Fatty acid profiling on gas chromatography–mass spectrometry (GC–MS) platforms is typically performed offline by manually derivatizing and analyzing small batches of samples. A GC–MS system with a fully integrated robotic autosampler can significantly improve sample handling, standardize data collection, and reduce the total hands-on time required for sample analysis. In this study, we report an optimized high-throughput GC–MS-based methodology that utilizes trimethyl sulfonium hydroxide (TMSH) as a derivatization reagent to convert fatty acids into fatty acid methyl esters. An automated online derivatization method was developed, in which the robotic autosampler derivatizes each sample individually and injects it into the GC–MS system in a high-throughput manner. This study investigated the robustness of automated TMSH derivatization by comparing fatty acid standards and lipid extracts, derivatized manually in batches and online automatically from four biological matrices. Automated derivatization improved reproducibility in 19 of 33 fatty acid standards, with nearly half of the 33 confirmed fatty acids in biological samples demonstrating improved reproducibility when compared to manually derivatized samples. In summary, we show that the online TMSH-based derivatization methodology is ideal for high-throughput fatty acid analysis, allowing rapid and efficient fatty acid profiling, with reduced sample handling, faster data acquisition, and, ultimately, improved data reproducibility.

## 1. Introduction

Fatty acid profiling is a commonly applied analytical methodology in academic research, health care, and industrial production and is used in a variety of applications that span from analyzing metabolic biomarkers to tracking environmental pollutants [1,2,3,4,5,6,7,8]. Advances in robotic platforms and associated software have enabled the automation of many tasks that previously required repetitive and extensive manual labor [9,10,11,12,13]. In addition to reducing sample handling, the integration of automatic sample preparation has improved data reproducibility for large sample batches [13,14,15,16]. The development of automated metabolite preparation and profiling methodologies that reduce researcher handling while maintaining or improving data quality are in high demand. Thus, advancements in automated sample preparation methodologies are beneficial to both academic and industrial laboratories.

Gas chromatography–mass spectrometry (GC–MS) platforms are well established for fatty acid analysis and are widely used in lipidomic and metabolomic research [8,17,18,19,20,21,22,23,24,25,26,27,28]. GC–MS analysis of fatty acids requires the derivatization of these analytes into non-polar derivatives, such as fatty acid methyl esters (FAMEs) [29]. This transformation improves sample volatility and subsequent chromatographic separation of the individual fatty acids [30,31,32]. Lipid researchers use numerous derivatizing agents, and each derivatization process can vary significantly in the number of steps and time required for sample preparation before GC–MS acquisition. Additionally, many derivatization processes involve lengthy incubations at high temperatures that can potentially induce metabolite degradation [33]. Derivatizing reagents, such as *N*,*O*-Bis(trimethylsilyl)trifluoroacetamide (BSTFA), boron trifluoride (BF_3_), and anhydrous hydrogen chloride (HCl), require long heated incubation steps at temperatures as high as 95 °C [34,35,36]. Metabolic degradation has been observed at 100 °C. However, fatty acids and their representative FAMEs have been reported to be stable when exposed to temperatures higher than 300 °C [33,37,38]. HCl, BF_3_, and potassium hydroxide (KOH) require a second extraction after the initial derivatization [39]. M-(trifluoromethyl)phenyltrimethylammonium hydroxide (TMTFTH) derivatization commonly calls for an overnight incubation before analysis [40]. These additional steps result in a high level of sample handling and time dedicated to prepare samples for fatty acid profiling compared to alternative metabolomic and lipidomic techniques that do not require derivatization. Additionally, without recourse to multiple analytical platforms analyzing samples simultaneously, extensive sample preparation leads to varying incubation times for individual samples. These factors reduce data reproducibility.

The automation of sample preparation is a viable method to eliminate researcher handling-induced variability. Similar approaches in automating sample preparation and derivatization have improved reproducibility in the analysis of sugars, organic acids, and amino acids [13,15,22,41]. In this study, we introduce a novel high-throughput automated GC–MS-based methodology that is ideal for uninterrupted fatty acid analysis. Trimethyl sulfonium hydroxide (TMSH), a derivatization reagent with a simple derivatization reaction and minimal sample handling that has been previously applied to fatty acid profiling [42,43,44,45,46], was selected and applied to work in an automated high-throughput manner. Capitalizing on the robotic autosampler attached to a GC–MS system, an automated online derivatization method was developed. The robotic sampler independently derivatizes each sample and injects it in an online high-throughput manner. In the developed automated method, each sample was derivatized immediately before injection in direct comparison with the common practice of manual batch derivatization followed by batch analysis, which results in time discrepancies between derivatization and injection for each sample. This study tested the robustness of the GC–MS method by comparing the automated and manual TMSH derivatization performed in batches or performed on the same set of batches.

## 2. Results and Discussion

### 2.1. Adaptation of Method to Automation

**Figure 1:**
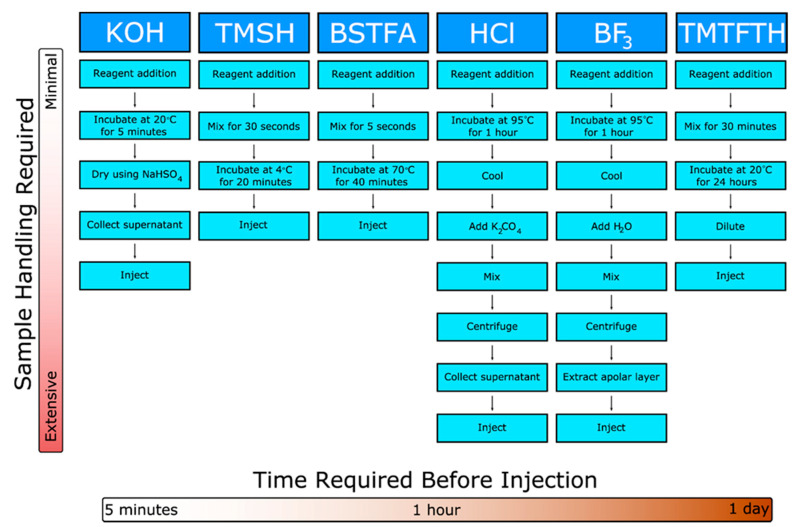
Time-based comparison of common fatty acid derivatization methodologies. Step-by-step workflow of common fatty acid derivatization techniques reported by various groups. Each independent step in sample preparation is illustrated, and the time required to prepare individual samples increases from left to right across the workflow. Required sample handling increases from top to bottom down each derivatization technique.

Commonly utilized fatty acid derivatization methods were compared (Figure 1), including the number of handling steps required for each derivatization reagent and the time before the sample is ready for injection and analysis. Several widely used derivatization reagents for fatty acids require extensive handling in the forms of secondary extractions or long heated incubations, such as those required by the standard KOH, HCl, and BF_3_ derivatization reactions.

**Figure 2:**
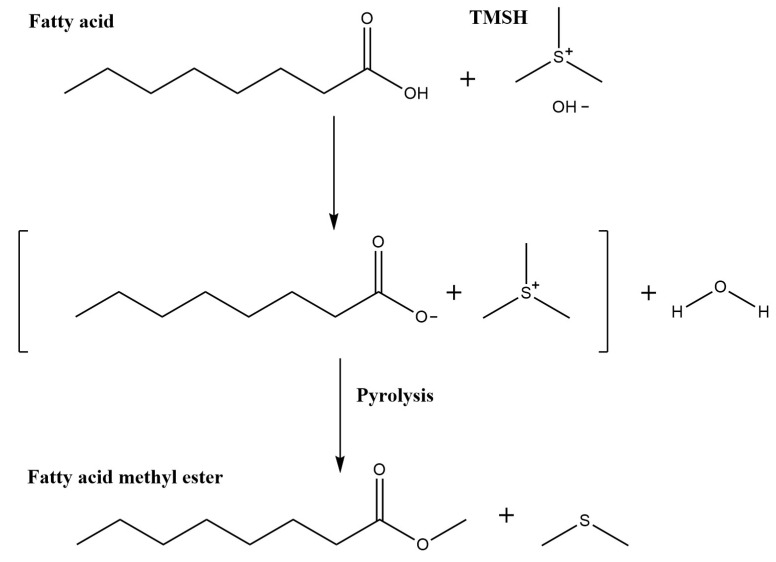
TMSH reaction outline for the conversion of fatty acids into fatty acid methyl esters by base-catalyzed transesterification and pyrolysis.

Due to the lack of need for a secondary extraction step and the short incubation time and simplicity, the TMSH derivatization method was selected and adapted for the Triplus RSH autosampler through the editor software. TMSH methylation occurs through a base-catalyzed transesterification reaction. Figure 2 shows the mechanism in which TMSH induces O-methylation of the carboxyl group of fatty acids when heated by the pyrolytic conditions of a GC injection port [47,48,49]. When performed manually, researchers can have a batch of samples ready for analysis within 30 min. However, as the samples are prepared in batches, the length of time each sample is incubated for can vary greatly between the first and last injected samples. Regarding the method outlined above, with batches of eight samples and a 20 min GC–MS runtime, there is a 160 min difference in incubation times between the first and last samples analyzed. In contrast, the automated method prepares each sample immediately before injection, and it is programmed to begin preparing each sample during the analysis of the previously injected sample. Automated sample handling ensures a consistent incubation period among all analyzed samples and reduces the variability between samples [15,22].

**Figure 3:**
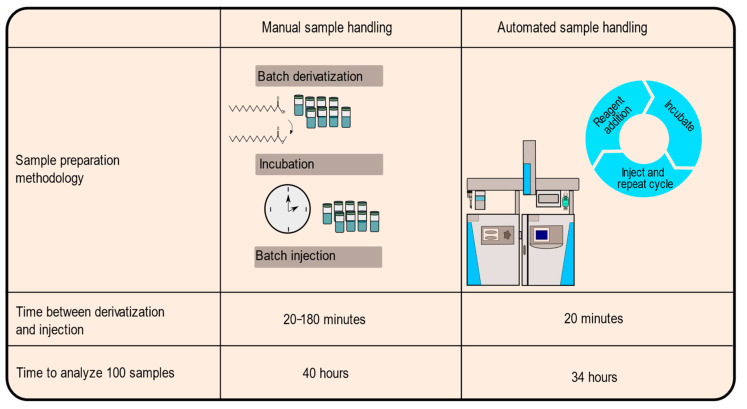
Reduction in time needed for analysis. Time-based comparison of manual and automated TMSH derivatization of fatty acids. Complete analysis of 100 samples manually in 10 batches of 10 samples each would require 40 h if batches were derivatized as soon as the previous batch finished. Automated derivatization would require 34 h with continuous derivatization and injection.

Moreover, automated derivatization decreases the time needed to analyze large batches of samples. Figure 3 depicts how automated derivatization reduces the time needed to analyze samples. To manually derivatize and analyze 100 samples in the manner presented above would require a minimum of 40 h just for sample derivatization and acquisition. In comparison, an analysis of 100 samples would take only 34 h if the autosampler derivatized and injected the samples in an online manner. Automated sample handling produces a 15% reduction in total time, including handling time by researchers, to derivatize and analyze the samples. A 15% reduction in analysis time itself may not represent a compelling improvement on its own; however, automated sample derivatization improves several additional aspects of sample handling and data acquisition. Primarily, automation reduces the negative consequences of researcher interaction required to acquire sample data, mainly through decreasing human errors that can be caused by fatigue or variability between researcher efforts. The robotic autosampler does not tire after 10 samples and has a higher consistency in the small tasks needed to prepare samples for analysis. It is important to consider that this methodology, although tested on a GC/single quad MS system, can be easily adapted to any analytical platform that has a programmable robotic autosampler.

### 2.2. Automated TMSH Derivatization Test on Fatty Acid Standard Mixture

**Figure 4:**
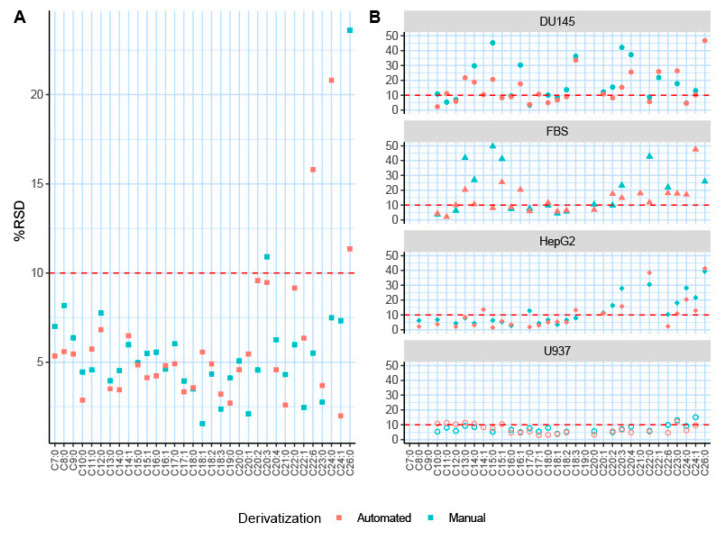
The %RSD comparison for fatty acid derivatization methods. Plot comparison of all %RSD values for each derivatization technique and each biological matrix. The red line across the plot at 15 represents a commonly accepted cutoff for highly reproducible metabolomics data. (**A**) The %RSD values for the 33 fatty acid standards utilized to validate the method. (**B**) The reproducibility of each fatty acid in the following biological matrices: human prostate cancer cell line DU145; fetal bovine serum (FBS); human liver cancer cell line HepG2; and U937, a human myeloid leukemia cell line.

**Figure 5:**
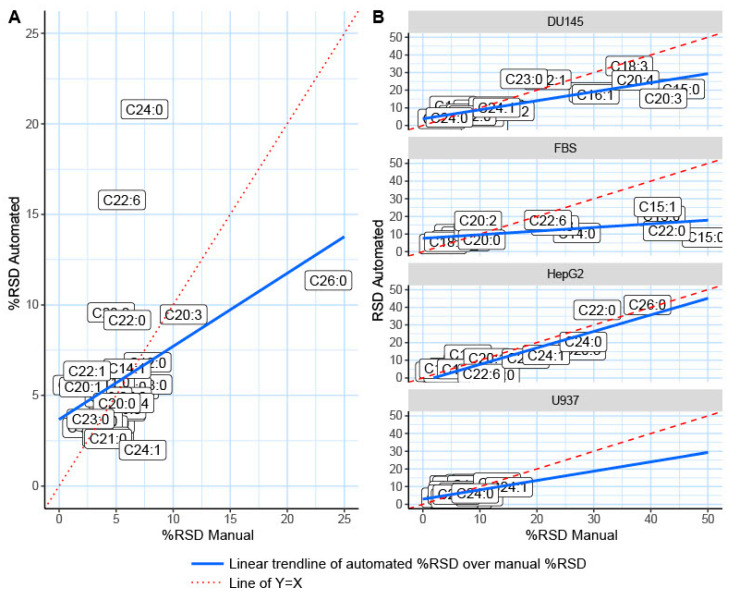
Automated derivatization improves %RSD trend (**A**). The ratio of the %RSD from the automatically derivatized fatty acid standards plotted over the %RSD of the manually derivatized fatty acid standards. The dotted red line represents a slope of 1 across the plot. Fatty acids that lie beneath that line demonstrate improved automation %RSD values. The blue line is a linear trendline derived from the data points. (**B**) The fatty acid derivatization ratios for all biological matrices (human prostate cancer cell line DU145, FBS, human liver cancer cell line HepG2, and human myeloid leukemia cell line) were tested.

To determine the efficiency of the automated TMSH derivatization, a mixture of 33 fatty acid standards was derivatized both manually and automatically. The reproducibility of the fatty acid derivatization is reported by the relative standard deviation (RSD) of each detected and confirmed lipid (Table 1); %RSD is a common indicator for the reproducibility of metabolomic data [13,22]. As previously reported, for a GC–MS analysis, a %RSD < 20% is considered acceptable reproducibility, whereas a %RSD < 10% is classified as superb reproducibility [13,20,50]. All except one of the fatty acid standards were detected and reported with good reproducibility (%RSD < 20%). Hexacosanoic acid (C26:0) exhibited a reproducibility of 23.61% using manual TMSH derivatization methodology. In total, 19 out of the 33 fatty acid standards utilized demonstrated improved reproducibility after automated derivatization when compared directly to manually derivatized standards (Figure 4A). Moreover, 31 out of the 33 fatty acid standards resulted in very high reproducibility (%RSD < 15%), as shown by the red dotted line (Figure 4A), after automated derivatization and online injection. Docosahexaenoic (C22:6) and tetracosanoic (C24:0) acids demonstrated a %RSD greater than 10% (13.65% and 12.58%, respectively) following the automated derivatization. Only docosahexaenoic and tetracosanoic acids exhibited worse reproducibility after being derivatized through automation, with no significant differences in reproducibility of the other fatty acid standards. The overall efficacy of automated derivatization is shown in Figure 5A, displaying the trend that automated derivatization has on %RSD across the fatty acid standards. When the automatically derivatized fatty acid %RSD values are plotted against the manually derived fatty acids, the trendline slope is less than one, indicating an overall improvement in %RSD and data reproducibility through automated derivatization.

### 2.3. Validation of the Automated TMSH Derivatization with Biological Samples

**Figure 6:**
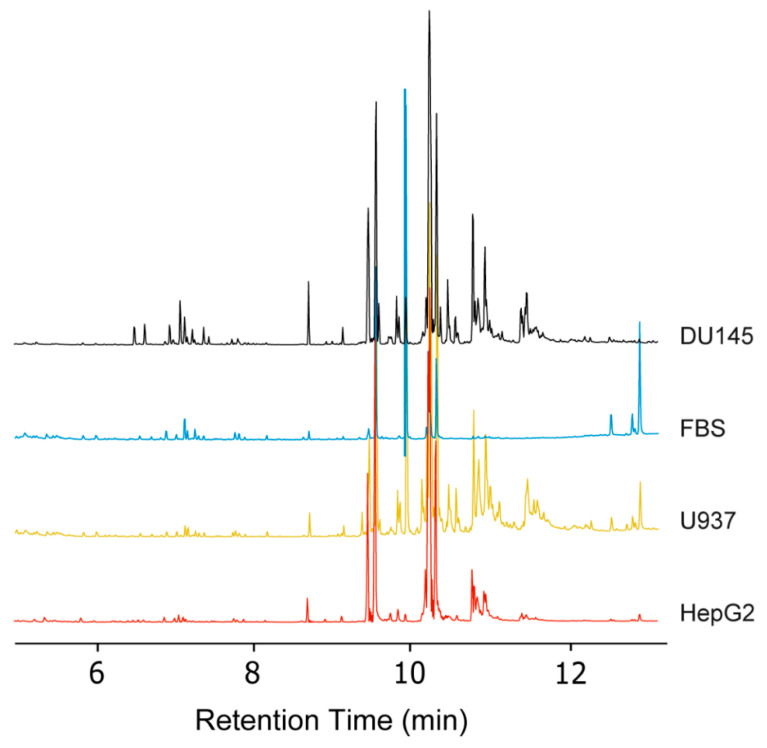
Chromatographs of FAMEs generated by TMSH derivatization. Representative chromatograms of biological matrices generated by TMSH derivatization, and the methods outlined in this paper.

After the automated TMSH derivatization method was successfully applied to the analysis of fatty acid standards, we tested this methodology using common biological matrices (Figure 4B and Figure 5B). Fatty acid profiling has numerous applications, such as disease detection, microbiome identification, and pollution monitoring [2,28,51,52]. The non-polar extracted fractions of HepG2, DU145, U937 cell samples, and FBS samples were analyzed. Figure 6 illustrates representative chromatograms for each sample type. The %RSD values are reported to represent reproducibility and are compared between manual and automated TMSH derivatization (Table 2). The %RSD values show a direct comparison for the %RSD values of each fatty acid and the trends that automation has upon reproducibility (Figure 4B and Figure 5B). Upon automation, the reproducibility of 17 out of the 33 fatty acids in the FBS samples improved. In terms of cell samples, automated derivatization improved the reproducibility of 9 fatty acids in the liver cancer cell line HepG2, 19 in the prostate cancer line DU145, and 15 in the acute myeloid leukemia line U937. Polyunsaturated fatty acids displayed higher variance than mono-unsaturated and saturated fatty acids in biological samples. Icosatetraenoic acid (C20:4) and eicosatrienoic acid (C20:4) demonstrated some of the highest variances in automated and manual TMSH derivatization when analyzed in DU145. Automation improved reproducibility by increasing %RSD from 71.96% to 28.56% and 40.73% to 20.70%. Overall, there is a definitive trend of improved %RSD values across all biological matrices, as shown in Figure 5B. The trendlines generated by the ratios of automated derivatization %RSD values over the manually derivatized %RSD values have slopes less than one, indicating that automated TMSH derivatization improved reproducibility over those of manually derivatized fatty acids in both standard mixture and biological samples.

## 3. Materials and Methods

### 3.1. Chemicals

Chemicals were purchased at the highest high-performance liquid chromatography (HPLC) purity grade available. HPLC-grade methanol, iso-octane, and methyl tert-butyl ether (MTBE) were purchased from Thermo Fisher Scientific (Waltham, MA). The TMSH solution was purchased from Sigma-Aldrich (St. Louis, MO, USA). The antioxidant 2,6-Di-tert-butyl-4-methylphenol was purchased from Acros Organics (Geel, Belgium).

Analytical grade standards of fatty acids from 7 carbons of length to 26 were purchased to test the efficacy of TMSH derivatization in both automated and manual methodologies. Heptanoic (C7:0), nonanoic (C9:0), decanoic (C10:0), undecanoic (C11:0), tridecanoic (C13:0), pentadecanoic (C15:0), (*Z*)-hexadec-9-enoic(C16:1), heptadecanoic (C17:0), (6*Z*, 9*Z*, 12*Z*)-octadeca-6,9,12-trienoic (C18:3), (9*Z*, 12*Z*)-octadeca- 9,12-dienoic (C18:2), (*Z*)-octadec-9-enoic (C18:1), nonadecanoic (C19:0), (5*Z*, 8*Z*, 11*Z*, 14*Z*, 17*Z*)-icosa-5,8,11,14,17-pentaenoic (C20:5), (5*Z*, 8*Z*, 11*Z*, 14*Z*)-icosa-5,8,11,14-tetraenoic (C20:4), heinecosanoic (C21:0), (4*Z*, 7*Z*, 10*Z*, 13*Z*, 16*Z*, 19*Z*)-docosa-4,7,10,13,16,19-hexaenoic (C22:6), (13*Z*, 16*Z*)-docosa- 13,16-dienoic (C22:2), (*Z*)-docos-13-enoic (C22:1), tricosanoic (C23:0), (*Z*)-tetracos-15-enoic (C24:1), tetracosanoic (C24:0), and hexacosanoic (C26:0) acid standards were acquired from Supelco through Sigma-Aldrich (St. Lious, MO). Dodecanoic (C12:0), tridecanoic (C13:0), 9(*Z*)-tetradecenoic (C14:1), tetradecanoic (C14:0), 10(*Z*)-pentedecenoic (C15:1), pentadecanoic (C15:0), (9*Z*)-hexadecenoic (C16:1), hexadecenoic (C16:0), 10(*Z*)-heptadecenoic (C17:1), heptadecanoic (C17:0), (6*Z*, 9*Z*, 12*Z*)-octadeca-6,9,12-trienoic (C18:3), (9*Z*, 12*Z*)-octadeca-9,12-dienoic (C18:2), (*Z*)-octadec-9-enoic(C18:1), octadecanoic (C18:0), 11(*Z*),14(*Z*),17(*Z*)-eicosatrienoic (C20:3), 11(*Z*),14(*Z*)-eicosadienoic (C20:2), 13(*Z*)-eicosenoic (C20:1), eicosanoic (C20:0), and docosanoic (C22:0) acid standards were acquired from Cayman Chemical (Ann Arbor, MI, USA). Octanoic acid was acquired from MP Biochemicals (Irvine, CA, USA). All standards were injected and analyzed at a concentration of 110 µg/mL.

### 3.2. Biological Samples

To validate this methodology, fatty acid standards and several sample matrices commonly used in research and clinical laboratories were derivatized, including three cell line extracts and characterized fetal bovine serum (FBS; Fischer Scientific, Hampton, NH, USA).

Three human cancer cell lines from the American Type Culture Collection (ATCC, Manasas, VA, USA) were selected to validate the derivatization method’s efficacy on laboratory samples. The hepatocyte cancer cell line HepG2 was grown in Eagle’s Minimum Essential Media (EMEM). The prostate and acute myeloid leukemia human cancer cell lines DU145 and U937 were cultured using Roswell Park Memorial Institute (RPMI) 1640 media. The cell culture media were modified to contain a 2 mM glutamine final concentration and 10% FBS. Cell growth was maintained at 37 °C and 5% CO_2_. HepG2 cells were harvested when the cell count reached 30 million cells. The total cell extract was split into ten equal aliquots with 3 million cells in each aliquot. Cells were harvested and washed three times with ice-cold phosphate buffer saline. Excess liquid was removed as much as possible before the cell pellets were snap frozen in liquid nitrogen. DU145 and U937 non-polar extracts were pooled and aliquoted into 12 and 16 equal aliquots, respectively, with the cell counts being 30 and 50 million, respectively. All samples were stored at −80 °C until analysis.

### 3.3. Lipid Extraction

Previously frozen sera and cell pellets were thawed on ice before extraction, and the whole extraction process was performed at 4 °C. The extraction of lipid molecules was accomplished by a modified Folch extraction that utilized HPLC-grade iso-octane as the non-polar phase instead of chloroform [24,53]. For serum samples, 50 μL of serum was combined with 100 μL of 0.9% saline and 200 μL of HPLC-grade methanol, followed by 20 μL of 1N HCl acid. Then, 20 μL of internal standard deuterated heptadecanoic acid (C17:0) was added for a final concentration of 20 ug/mL, and 10 μL of 200 ug /mL of the antioxidant butylated hydroxytoluene (BHT) was added to the mixture to reach a final polar volume of 400 μL. Finally, 800 μL of HPLC-grade iso-octane was added to each sample and vortexed for 30 s [54,55]. After vortex mixing, samples were shaken for 10 min at 2500 rpm on a Heidolph Vibramax 110 (Schwabach, Germany). Immediately after shaking, samples were centrifuged at 4750 rpm for 20 min at a temperature of 4 °C. The non-polar iso-octane supernatant was recovered using 500 µL glass Hamiltonian syringes and transferred to a new 1.5 mL glass vial. These non-polar fractions were dried for 1.5 h at 4 °C in a Labconco Refrigerated Centrivap Concentrator coupled with Labconco −84 °C Centrivap Cold Trap (Kansas City, MO, USA). Dried samples were stored at −80 °C until analysis.

### 3.4. Manual Derivatization

The use of TMSH for manual derivatization was adapted from previously published methods with slight modifications [43,56]. Previously dried samples were grouped into batches of 6–8 samples. Each batch was derivatized manually by the addition of 30 μL TMSH and 60 μL MTBE into each sample vial. Each vial was vortexed for 30 s to achieve adequate mixing of reagents and then placed in the temperature-controlled sample tray attached to the Triplus RSH autosampler (Thermo Scientific, Waltham, MA, USA). Samples were set to incubate for 20 min inside the sample tray and maintained at a constant 4 °C. After the 20 min incubation, the TriPlus RSH autosampler arm injected 3 µL of each sample for analysis. FBS and HepG2 samples required an increase in the injection volume to achieve adequate signal stability. Each batch included an empty vial containing the mixed derivatizing agents to monitor for reagent contamination and account for reagent background readings.

### 3.5. Automated Derivatization

The Thermo Scientific Triplus RSH Sampling Workflow Editor Software (Thermo Scientific, Waltham, MA, USA) was used to program the automated derivatization method. The workflow editor allows for the adaptation of various modules and programmable steps to create an easily modifiable method to handle, derivatize, and inject samples in an online manner. This automated method utilizes the robotic Triplus RSH Autosampler to add reagents, vortex, and inject samples in an automated and online manner. QSertVials with fused glass inserts that contained dried samples were placed in the refrigerated sample box attached to the autosampler. The normal polytetrafluoroethylene (PTFE) screw caps were replaced with similar caps that contained a magnetic covering, enabling automated movement in conjunction with the magnetic syringe adaptor of the autosampler. A 100 µL airtight syringe was used to add 60 µL of MTBE to a sample vial. The syringe was then rinsed three times with 80 µL of Optima HPLC-grade methanol before 30 µL of TMSH was added to the sample vial. The syringe was rinsed again before the sample vial was moved to the attached vortex module. The sample vial was vortexed for 30 s at 1500 rpm and then returned to the refrigerated sample tray and incubated for 20 min at 4 °C. The 100 µL syringe was undocked and replaced with a 10 µL syringe used to inject up to 3 µL of the derivatized sample into the GC–MS for immediate analysis.

### 3.6. GC–MS Setup and Analysis

The GC–MS system utilized is a Thermo Scientific Trace 1310 GC (Thermo Fisher Scientific, Waltham, MA, USA) coupled to an ISQ single quadrupole MS. The Triplus RSH autosampler is mounted to the system with several attached modules, including a temperature-controlled drawer for sample storage, a mounted vortex unit capable of mixing several sized sample vials, two large reagent reservoirs capable of containing 100 mL of chemical reagents or washing solutions, and an automated tool change (ATC) station to facilitate the use of multiple syringes. To enable the transport of sample vials between modules for automated derivatization, 9 mm magnetic AVCS screw caps from Thermo Scientific were needed and were used to seal the sample vials that undergo automated derivatization. Then, 3 µL of each derivatized sample was injected into the GC–MS by the Triplus RSH autosampler. Samples were injected into a 4 mm glass split/splitless liner packed with quartz wool. The inlet was maintained at 250 °C, with a split ratio of 33.3 and a split flow of 55.0 mL/min into a Phenomenex (Torrance, CA, USA) Zebron ZB 1-ms column. The column dimensions were the following: 30 m long, a 0.25 mm inner diameter, and a 0.25 µm dimethylpolysiloxane stationary phase. Ultra-high purity helium from Praxair (Austin, TX, USA) was used as a carrier gas at a flow rate of 1.10 mL/min, and injection port septa were changed every 50 runs to minimize contamination between samples. The GC oven was held at the starting temperature of 80 °C for two minutes and then set to increase 20 °C/min to 220 °C and held there for 1 min before resuming the previous rate of 20 °C/min to a final temperature of 300 °C. The oven was retained at 300 °C for five minutes to clean the column of residual cholesterol and its derivatives. The transfer line to the ISQ mass detector was kept at 280 °C, and the ion source was maintained at 230 °C. Ion scanning began 2.5 min after the initial injection and acquired ions in the mass range of 50–500 amu with a scan time of 0.5 s. Ionization was accomplished through electron impact at 70 eV.

Individual fatty acids were confirmed by their corresponding standards’ fragmentation patterns and retention times. The fragmentation pattern retention times were used to create a component-based processing method to extract ion areas for each confirmed fatty acid. Individual ion peak features were extracted by Xcalibur version 4.4, which utilized the ICIS peak detection algorithm with minimal smoothing and a maximum baseline of 10 scans to integrate the standard confirmed fatty acid peaks. Extracted ion peak area data were extracted and processed with MATLAB and R statistical functions to calculate averages, standard deviations, and relative standard deviations for each fatty acid and derivatization method reported below.

## 4. Conclusions

Previous studies automating metabolite extractions and derivatization have reported improved efficiency and reproducibility for metabolite analysis [12,16,22,57,58]. The advent of method editor software enables the rapid adaptation of simple sample preparation methods, such as the TMSH derivatization method detailed in this study. Automated TMSH derivatization reduced (i) researcher handling of samples and potential errors associated with manual derivatization handling and (ii) the total time needed for the analysis. Additionally, the data obtained from the automated TMSH derivatization of analytical standards showed improved data reproducibility compared to manually handled samples. When applied to biological matrices, the automated TMSH derivatization demonstrated similar reproducibility on most fatty acids analyzed across several cancer cell lines and serum samples. This automated derivatization methodology, although validated on a GC–MS system, could be implemented on any analytical platform with autosampler and vortex modules. This study presents the application and adaptation of a well-established fatty acid derivatization technique to be performed in an automated and online manner, reducing researcher-induced variability and improving data reproducibility while enabling high-throughput fatty acid profiling.

## Figures and Tables

**Table 1:** Compiled relative standard deviations of analyzed free fatty acid standards after manual and automated TMSH derivatization. Improvements in reproducibility are indicated with bolded RSD values.

Fatty Acid	HMDB ID	Carbons: Double Bonds	FAME Molecular Mass	Quantifier Ion (*m*/*z*)	Standards n = 6	
					Manual	Automatic
Heptanoic acid	HMDB0000666	C7:0	144.21	73.98	7.00	5.35
Octanoic acid	HMDB0000482	C8:0	158.24	73.98	8.18	5.59
Nonanoic acid	HMDB0000847	C9:0	172.26	73.98	6.36	5.46
Decanoic acid	HMDB0000511	C10:0	186.27	73.98	4.45	2.88
Undecanoic acid	HMDB0000947	C11:0	200.29	73.95	4.57	5.73
Dodecanoic acid	HMDB0000638	C12:0	214.32	73.93	7.76	6.82
Tridecanoic acid	HMDB0000910	C13:0	228.35	73.94	3.96	3.51
9(*Z*)-Tetradecenoic acid	HMDB0002000	C14:1	240.36	55.05	5.97	6.48
Tetradecanoic acid	HMDB0000806	C14:0	242.38	73.99	4.53	3.45
9(*Z*)-Pentedecenoic acid	HMDB0029765	C15:1	254.41	55.05	5.49	4.13
Pentadecanoic acid	HMDB0000826	C15:0	256.42	73.96	4.97	4.85
9(*Z*)-Hexadecenoic acid	HMDB0003229	C16:1	268.41	55.05	4.61	4.81
Hexadecanoic acid	HMDB0000220	C16:0	270.45	73.95	5.56	4.24
10(*Z*)-Heptadecenoic acid	HMDB0060038	C17:1	282.46	55.05	3.94	3.33
Heptadecanoic acid	HMDB0002259	C17:0	284.46	73.96	6.03	4.91
(6*Z*, 9*Z*, 12*Z*)-Octadecatrienoic acid	HMDB0003073	C18:3	292.44	292.18	2.37	3.21
(9*Z*, 12*Z*)-octadecadienoic acid	HMDB0000673	C18:2	294.45	294.18	4.33	4.90
9(*Z*)-octadecenoic acid	HMDB0000207	C18:1	296.68	296.20	1.55	5.57
Octadecanoic acid	HMDB0000827	C18:0	298.48	73.98	3.50	3.57
Nonadecanoic acid	HMDB0000772	C19:0	312.53	73.96	4.11	2.71
(5*Z*, 8*Z*, 11*Z*, 14*Z*)-Icosatetraenoic acid	HMDB0001043	C20:4	318.49	318.20	6.26	4.57
(5*Z*, 8*Z*,11*Z* )-Eicosatrienoic acid	HMDB0010378	C20:3	320.51	320.20	10.91	9.47
(11*Z*, 14*Z*)-Eicosadienoic acid	HMDB0005060	C20:2	322.51	322.25	4.56	9.58
13(*Z*)-Eicosenoic acid	HMDB0035159	C20:1	324.50	324.19	2.10	5.45
Eicosanoic acid	HMDB0002212	C20:0	326.56	73.99	5.07	4.57
Heinecosanoic acid	HMDB0002345	C21:0	340.58	73.97	4.30	2.60
(4*Z*, 7*Z*, 10*Z*, 13*Z*, 16*Z*, 19*Z*)-Docosahexaenoic acid	HMDB0002183	C22:6	342.52	79.06	5.51	13.65
(13*Z*)-Docosenoic acid	HMDB0002068	C22:1	352.57	55.05	2.46	6.35
Docosanoic acid	HMDB0000944	C22:0	354.59	74.00	5.97	9.16
Tricosanoic acid	HMDB0001160	C23:0	368.62	74.00	2.76	3.70
(15*Z*)-Tetracosenoic acid	HMDB0002368	C24:1	379.62	55.06	7.33	1.99
Tetracosanoic acid	HMDB0002003	C24:0	381.36	73.99	7.49	12.58
Hexacosanoic acid	HMDB0002356	C26:0	410.69	73.96	23.61	4.27

**Table 2:** Compiled relative standard deviations of analyzed free fatty acids in biological matrices after manual and automated TMSH derivatization. Improvements in reproducibility are indicated with bolded %RSD values. NF = not found.

Fatty Acid	Carbons:Double Bonds	HepG2 n = 6		DU145 n = 6		U937 n = 6		FBS n = 6	
		Manual	Automatic	Manual	Automatic	Manual	Automatic	Manual	Automatic
Heptanoic acid	C7:0	NF	NF	NF	NF	NF	NF	NF	NF
Octanoic acid	C8:0	12.29	6.74	10.80	5.16	7.88	5.33	24.24	18.95
Nonanoic acid	C9:0	NF	NF	NF	NF	NF	NF	NF	NF
Decanoic acid	C10:0	4.77	4.79	10.36	2.23	3.86	4.76	7.32	4.87
Undecanoic acid	C11:0	4.13	3.68	NF	NF	NF	NF	6.52	5.87
Dodecanoic acid	C12:0	3.33	2.55	5.29	2.94	3.15	4.48	7.18	5.63
Tridecanoic acid	C13:0	5.44	7.24	NF	NF	14.27	11.59	21.36	23.31
9(*Z*)-Tetradecenoic acid	C14:1	NF	NF	NF	NF	NF	NF	NF	NF
Tetradecanoic acid	C14:0	5.23	5.43	NF	NF	3.02	4.06	8.70	5.09
9(*Z*)-Pentedecenoic acid	C15:1	3.81	8.69	51.44	5.88	6.97	3.00	5.50	12.39
Pentadecanoic acid	C15:0	6.17	7.41	41.89	21.60	3.13	4.66	11.34	7.35
9(*Z*)-Hexadecenoic acid	C16:1	3.37	3.19	30.09	17.32	2.26	1.82	19.98	7.27
Hexadecanoic acid	C16:0	3.25	5.00	7.93	11.17	2.19	3.28	6.83	5.04
10(*Z*)-Heptadecenoic acid	C17:1	4.79	14.33	NF	NF	4.51	3.04	9.08	9.14
Heptadecanoic acid	C17:0	6.49	3.79	19.65	7.05	1.00	1.03	4.69	2.69
(6*Z*, 9*Z*, 12*Z*)-Octadecatrienoic acid	C18:3	NF	NF	NF	NF	14.21	19.39	NF	NF
(9*Z*, 12*Z*)-octadecadienoic acid	C18:2	NF	NF	NF	NF	5.42	2.74	20.74	5.43
9(*Z*)-octadecenoic acid	C18:1	1.26	6.18	8.15	5.88	1.25	1.28	15.88	4.60
Octadecanoic acid	C18:0	9.03	2.78	11.31	7.59	1.79	2.04	5.28	4.33
(5*Z*, 8*Z*, 11*Z*, 14*Z*)-Icosatetraenoic acid	C20:4	14.34	15.46	71.96	28.56	9.21	3.77	NF	NF
(5*Z*, 8*Z*, 11*Z*)-Eicosatrienoic acid	C20:3	11.10	15.08	40.73	20.70	10.79	5.57	NF	NF
(11*Z*, 14*Z*)-Eicosadienoic acid	C20:2	7.70	12.23	18.63	12.29	NF	NF	NF	NF
13(*Z*)-Eicosenoic acid	C20:1	6.19	12.72	11.22	4.70	7.81	4.96	NF	NF
Eicosanoic acid	C20:0	27.20	5.50	10.44	4.78	5.07	3.46	44.30	29.24
Heinecosanoic acid	C21:0	NF	NF	NF	NF	NF	NF	NF	NF
(4*Z*, 7*Z*, 10*Z*, 13*Z*, 16*Z*, 19*Z*)-Docosahexaenoic acid	C22:6	22.28	14.46	NF	NF	6.48	5.94	NF	NF
(13*Z*)-Docosenoic acid	C22:1	NF	NF	11.85	9.28	NF	NF	NF	NF
Docosanoic acid	C22:0	8.70	20.28	11.27	3.11	5.10	6.38	28.19	17.57
Tricosanoic acid	C23:0	NF	NF	47.77	14.83	8.81	6.36	NF	NF
(15*Z*)-Tetracosenoic acid	C24:1	8.30	14.30	12.74	6.92	8.66	3.37	42.72	6.93
Tetracosanoic acid	C24:0	6.59	33.25	4.77	4.48	8.20	2.44	44.78	7.33
Hexacosanoic acid	C26:0	10.07	7.00	8.50	7.60	6.25	3.08	11.95	7.41

## Data Availability

Compiled data is reported in the tables above. The raw data files are available from authors upon request.

## Sample Availability

Samples of the compounds are not available from the authors. Fatty acid standards are available from the manufacturers listed in the materials and methods section.
